# Novel Combinations of Human Immunomodulatory mAbs Lacking Cardiotoxic Effects for Therapy of TNBC

**DOI:** 10.3390/cancers14010121

**Published:** 2021-12-27

**Authors:** Cinzia Vetrei, Margherita Passariello, Guendalina Froechlich, Rosa Rapuano Lembo, Emanuele Sasso, Nicola Zambrano, Claudia De Lorenzo

**Affiliations:** 1 Ceinge—Biotecnologie Avanzate s.c.a.r.l., Via Gaetano Salvatore 486, 80145 Naples, Italy; cinzia.vetrei@unina.it (C.V.); margherita.passariello@unina.it (M.P.); froechlich@ceinge.unina.it (G.F.); rosa.rapuano@unimi.it (R.R.L.); emanuele.sasso@unina.it (E.S.); zambrano@unina.it (N.Z.); 2 Department of Molecular Medicine and Medical Biotechnology, University of Naples “Federico II”, Via Pansini 5, 80131 Napoli, Italy; 3 European School of Molecular Medicine, University of Milan, 20122 Milan, Italy

**Keywords:** TNBC, PD-L1, mAbs, immunotherapy, cardiotoxicity

## Abstract

**Simple Summary:**

Immunotherapy has revolutionized the management of cancer by improving outcomes of triple-negative breast cancer (TNBC). Recently, programmed death-ligand 1 (PD-L1), was identified as a target for TNBC and several preclinical and clinical trials are currently focusing on combinatorial treatments of immunomodulatory mAbs with chemotherapy, radiotherapy, or other mAbs. Here, we tested in in vitro models novel combinations of immunomodulatory mAbs on TNBC cell lines and on cardiomyocytes, in comparison with the mAbs approved by FDA for cancer therapy, in order to identify at early stages the more potent anti-cancer combinations endowed with low or no cardiotoxic side effects.

**Abstract:**

Triple-negative breast cancer (TNBC) is a highly aggressive subtype of breast cancer characterized by a higher mortality rate among breast cancer subtypes. Poly(ADP-ribose) polymerase (PARP) inhibitors are used in clinics to treat a subgroup of TNBC patients, but other targeted therapies are urgently needed. Programmed death-ligand 1 (PD-L1), involved in tumor immune escape, was recently identified as a target for TNBC; accordingly, the anti-PD-L1 monoclonal antibody (mAb), atezolizumab, has been approved by FDA in combination with Paclitaxel for the therapy of metastatic TNBC. Here, we tested novel combinations of fully human immunomodulatory mAbs, including anti-PD-L1 mAbs generated in our laboratory and atezolizumab, on TNBC and other tumor cell lines. We evaluated their anti-tumor efficacy when used as single agents or in combinatorial treatments with anti-CTLA-4 mAbs in in vitro co-cultures of hPBMCs with tumor cells, by measuring tumor cell lysis and IL-2 and IFNγ cytokines secretion by lymphocytes. In parallel, by using co-cultures of hPBMCs and cardiomyocytes, we analyzed the potential cardiotoxic adverse side effects of the same antibody treatments by measuring the cardiac cell lysis and the secretion of pro-inflammatory cytokines. We identified novel combinations of immunomodulatory mAbs endowed with more potent anti-cancer activity on TNBC and lower cardiotoxic side effects than the combination of atezolizumab and ipilimumab.

## 1. Introduction

Breast cancer, the most commonly diagnosed cancer in women and the leading cause of cancer death [1], include up to 20 clinically different subtypes [2,3], each of them characterized by specific morphological traits and prognosis. Among them, triple-negative breast cancer (TNBC) is a particularly aggressive subtype of breast cancer, defined by the lack of expression of estrogen receptor (ER), progesterone receptor (PR), and human epidermal growth factor receptor 2 (HER2). TNBC accounts for about 10–20% of breast cancer cases and the affected patients show high risk of recurrence with a higher death rate than any other subtype. Due to the resistance to conventional chemotherapeutic drugs, TNBC is also characterized by a shorter time to recurrence and a worse overall survival (OS), compared to non-TNBC patients (approximately 18 months or less) [4,5,6,7,8]. The mainstay of treatments for TNBC patients is represented by the conventional chemotherapy; however, during the last decade the targeted therapy has revolutionized the management of cancer by improving also that of TNBC. Indeed, poly(ADP-ribose) polymerase (PARP) inhibitors are used in the clinics to treat a subgroup of TNBC patients bearing BRCA1 and BRCA2 mutations [9]. In particular, due to the higher expression of programmed cell death ligand-1 (PD-L1) on TNBC compared to the other cancer subtypes, the IMpassion130 Trial [10] combined nab-paclitaxel with the anti-PD-L1 atezolizumab, and demonstrated a remarkably prolonged progression-free survival (PFS) in patients with metastatic TNBC, leading to FDA approval for patients with unresectable advanced PD-L1-positive TNBC even though the following IMpassion131 trial (NCT03125092) caused the withdrawal of atezolizumab [11]. Atezolizumab is a humanized antibody which inhibits PD-1/PD-L1 interaction, inducing the activation of T cells [12,13]. Furthermore, considering that PD-L1 is also involved in tumor “immune escape”, and plays a role in tumor growth and proliferation by affecting mitogen-activated protein kinase (MAPK) pathway [14,15], atezolizumab and other anti-PD-L1 monoclonal antibodies (mAbs) can efficiently inhibit tumor growth by exploiting different mechanisms of action.

Since mAb-based monotherapy can only achieve about 10% response, several preclinical and clinical trials are currently focusing on combinatorial treatments with the aim to achieve higher therapeutic index with respect to monotherapy [16,17]. To date, combinatorial approaches of immunomodulatory mAbs with chemotherapy, radiotherapy, and other therapies are under evaluation also in TNBC, such as: trials based on the use of the anti-PD-1 mAb pembrolizumab in combination with the anti-microtubule agent eribulin [18], or with three different chemotherapy regimens comprising either nab-paclitaxel, paclitaxel or carboplatin plus gemcitabine [19], or in combination with niraparib (a selective inhibitor of PARP1/2 used in TOPACIO trial; phase 2) [20]. Furthermore, the TONIC trial (in phase 2) is based on treatment with the anti-PD-1 Nivolumab plus the cyclophosphamide-doxorubicin-cisplatin (CAP) chemotherapy regimen [21], whereas the COLET trial in phase 2, involves the anti-PD-L1 atezolizumab and the MEK1 inhibitor cobimetinib plus paclitaxel chemotherapy [22].

Recently, considering the benefits of combinatorial treatments of anti-PD-1 and anti-CTLA-4 monoclonal antibodies, such as nivolumab and ipilimumab, in many types of cancer [23,24] a small pilot study was carried out also on TNBC, highlighting a positive response in 16.7% of patients when they were treated with the anti-PD-L1 durvalumab and the anti-CTLA-4 mAb tremelimumab [25].

Unfortunately, combinatorial treatment of immunomodulatory mAbs can also induce a wide spectrum of immune-related adverse events (irAEs) [26,27,28], that in severe cases could lead to myocarditis, cardiomyopathy, pericarditis, and Takotsubo syndrome [29,30,31,32,33].

Herein, we tested on TNBC cell lines the effects of novel combinations of fully human immunomodulatory mAbs, previously generated in our laboratory [34], and compared them to those of clinically validated mAbs such as atezolizumab and ipilimumab by evaluating not only their anti-tumor efficacy, but also their potential side effects on cardiac cells.

## 2. Materials and Methods

### 2.1. Antibodies and Human Recombinant Proteins

The following antibodies were used: anti-CTLA-4 mAb ipilimumab (Yervoy, Bristol Myers Squibb, NY, USA); human anti-PD-L1 mAb atezolizumab (Creative Biolabs, Ramsey Road, Shirley, NY, USA); commercial anti-human PD-L1 human mAb (G&P Biosciences, Santa Clara, CA, USA); commercial human anti-CTLA-4 antibody (R&D Systems, Minneapolis, MN, USA); commercial anti-human LAG-3 monoclonal antibody(R&D Systems, Minneapolis, MN, USA) anti-human IgG (H + L) HRP-conjugate antibody (Promega, Madison, WI, USA); HRP-conjugated anti-human IgG (Fab’)2 goat monoclonal antibody (Abcam, Cambridge, UK); anti-actin antibody (Sigma-Aldrich, Darmdstadt, Germany); HRP-conjugated anti-human IgG (Fc-specific) and IgG HRP conjugate anti-Mouse (were from Sigma). ID1 (anti-CTLA-4) and PD-L1_1 (anti-PD-L1) monoclonals were produced and purified as previously described [34,35]. Briefly, mAbs were purified from conditioned media of HEK293ES_1 cells transfected with expression vectors encoding heavy and light chains. Supernatants were clarified by centrifugation at 3000 rpm for 20 min and purified by protein A affinity chromatography using HiTrap Protein A HP (17-0402-01) General Electric (Boston, MA, USA). Additional polishing steps consisted of buffer exchange in PBS by PD-10 desalting columns packed with Sephadex G-25 resin (17085101) Cytiva, Previously GE Healthcare (Boston, MA, USA) and sterilization by 0.2µm filtration by using Whatman Puradisc 4 syringe filters (WHA67910402) Millipore (Boston, MA, USA).

### 2.2. Cell Cultures

MDA-MB-231 breast cancer cells were cultured in Dulbecco’s modified Eagle’s medium (DMEM, Gibco, Life Technologies, Paisley, UK). BT-549 breast cancer cells were cultured in Roswell Park Memorial Institute 1640 Medium (RPMI 1640, Gibco, Life Technologies, Paisley, UK). A-549 lung cancer cells were cultured in Kaign’s modification of Ham’s F-12 medium (F-12K, American Type Culture Collection, Manassas, VA, USA). Human fetal cardiomyocytes were cultured in cardiac myocyte medium (CMM, Innoprot, Derio—Bizkaia, Spain). HuT-78 deriving from cutaneous T lymphocytes were cultured in Iscove’s modified Dulbecco’s medium (IMDM, Sigma-Aldrich, St. Louise, MO, USA). All the cell lines were purchased from the American Type Culture Collection (ATCC) and cultured in humidified atmosphere containing 5% CO_2_ at 37 °C. The media were supplemented with 10% (20% in the case of HuT-78 cells) heat-inactivated fetal bovine serum (FBS, Sigma, St. Louis, MO, USA) and were used after addition of 50 U/mL penicillin, 50 μg/mL streptomycin, 2 nM L-glutamine (all from Gibco, Life Technologies, Paisley, UK). Human peripheral blood mononuclear cells (hPBMCs) were thawed out from frozen samples previously obtained from normal donor buffy coats (Blood Bank of the Medical School of the University of Naples “Federico II”) as previously described [36].

### 2.3. Enzyme-Linked Immunosorbent Assays (ELISA)

To measure the cell surface expression level of LAG-3, CTLA-4, and PD-L1 proteins on tumor cells, cell ELISA assays were performed on breast (MDA-MB-231 and BT-549 cell lines), lung (A-549 cell line) and cells derived from cutaneous T cell lymphoma (HuT-78 cell line). As previously described [37], cells were plated in triplicates into a NuncTM round-bottom 96-well plate at the density of 2 × 10^5^ cells/well and incubated with a blocking solution (PBS/BSA 6%) for 20 min at RT. Then, a first incubation was performed in the absence or in the presence of anti-LAG-3 (R&D Systems), anti-CTLA-4 (R&D Systems) or anti-PD-L1 (G&P Biosciences) mAbs at a concentration of 200 nM in PBS/BSA 3% buffer solution for 2 h at RT with gentle agitation. After the incubation with the primary antibodies, the plates were washed with PBS and incubated with an appropriate HRP-conjugated antibody for 1 h at room temperature. Extensive washes were performed before 3,3′,5,5′-Tetramethylbenzidine (TMB) (Sigma-Aldrich, St. Louise, MO, USA) reagent was added for 10 min; then the reaction was quenched with an equal volume of 1 N HCl. Absorbance at 450 nm was measured by the Envision plate reader (Perkin Elmer, 2102, San Diego, CA, USA).

To perform the competitive assays, for determining whether the PD-L1_1 or the ID-1 mAb recognize different epitopes from those recognized by atezolizumab or ipilimumab, respectively, PD-L1- or CTLA-4/Fc chimeric protein was immobilized on 96-well plate (5 μg/mL). After blocking the plate was pre-incubated for 2 h with saturating concentrations of each unlabeled targeted mAb (400 nM) in 3% BSA in PBS in agitation at room temperature. After extensive washes with PBS, increasing concentrations of biotinylated atezolizumab or ipilimumab mAb were added and the binding detected by incubating the plates with HRP-conjugated streptavidin for 30 min in agitation at room temperature. The following washes and detection steps were performed as mentioned above.

### 2.4. Western Blotting Analyses

MDA-MB-231, BT-549 cells, and A-549 and HuT-78 cancer cells were plated at a density of 6 × 10^5^ cells/well and incubated for 72 h at 37 °C. Cells were scraped and centrifuged at 1200 rpm for 5 min; the cell pellets were lysed in a buffer containing 10 mM Tris-HCl (pH 7.4), 0.5% Nonidet-P-40, 150 mM NaCl and 1 mM Sodium orthovanadate (Sigma-Aldrich, St. Louis, MO, USA), in the presence of protease inhibitors (Roche, Indianapolis, IN, USA). After incubation on ice for 20 min, the cell extracts were clarified by centrifugation at 12,000 rpm for 15 min at 4 °C. Bradford colorimetric assays (Sigma-Aldrich, St. Louis, MO, USA) were performed to determine protein concentration and Western blotting analyses were carried out by incubating the membranes with the commercial anti-LAG-3 primary antibody (R&D Systems, Minneapolis, MN, USA) and anti-actin antibody (Sigma-Aldrich, Darmdstadt, Germany), followed respectively by goat anti-Mouse polyclonal (IgG-HRP conjugated) and by goat anti-rabbit polyclonal IgG (HRP-conjugated) secondary antibodies (both from Sigma).

### 2.5. Cell Viability by MTT Assays

In order to evaluate the effects induced by the immunomodulatory mAbs, cancer cells were plated at a density of 5 × 10^3^ cells/well in 96-well flat-bottom plates for 16 h. Then, they were incubated in the absence or in presence of atezolizumab, LAG-3_1, PD-L1_1 mAbs or their combinations at the concentrations of 100 nM, for 72 h. Cells untreated or treated with an unrelated control IgG were used as negative controls. After the incubation, the medium was removed and replaced by a new medium containing MTT (0.5 mg/mL final concentration). Cells were incubated for 4 h in a humidified atmosphere containing 5% CO_2_ at 37 °C, until intracellular purple formazan crystals were visible. Then, medium containing MTT was discarded and solubilizing solution (DMSO) was added. After 30 min of incubation in a humidified atmosphere containing 5% CO_2_ at 37 °C; absorbance was measured at 570 nm. Cell survival was expressed as percent of viable cells with respect to the untreated cells, used as negative control.

### 2.6. Cytotoxicity Assays and LDH Detection

Cells co-cultured with hPBMCs (effector:target ratio 5:1) were treated with the immunomodulatory mAbs used as single agents or in combination. Tumor cells were plated in 96-well flat-bottom plates at the density of 1 × 10^4^ cells/well, whereas HFC cardiac cells were plated at a density of 1.5 × 10^4^, for 16 h. Then, hPBMCs were added in the absence or presence of ipilimumab, atezolizumab, ID-1, PD-L1_1, or LAG-3_1 mAbs, used alone or in combinations at a concentration of 100 nM. Untreated cells and cells incubated with an unrelated IgG (100 nM) were used as negative controls. After the treatment (24–48 h), cell lysis was evaluated by detecting the level of lactate dehydrogenase (LDH) released by tumor or cardiac cells in the supernatant of co-cultures described above, by using LDH detection kit (Thermofisher Scientific, Rockford, IL, USA), following the manufacturer’s recommendations. Cell lysis was analyzed by measuring the fold increase of LDH in the presence of each treatment, with respect to that present in the supernatant of negative controls. Cytolysis values were obtained from at least three independent values.

### 2.7. Cytokine Secretion Assays

Supernatants of co-cultures of cells with hPBMCs were analysed by ELISA assays to evaluate the secretion of interleukin 6, interleukin-2, IFN-γ, and granzyme B. Briefly, after the treatments in the absence or presence of immunomodulatory mAbs, supernatants were centrifuged and treated for quantification of human IL-6 (ELISA MAXTM Deluxe Set Human IL-6, BioLegend, San Diego, CA, USA), human IL-2, IFN-γ, and granzyme B (DuoSet ELISA, R&D Systems, Minneapolis, MN, USA), according to the producer’s recommendations. ELISA assays to measure TNF-α cytokine level were performed by using Ella Automated Immunoassay system (R&D Systems, Minneapolis, MN, USA), following the producer’s recommendations. Concentration values were reported as the mean of at least three determinations.

### 2.8. Statistical Analyses

Error bars were calculated on the basis of the results obtained by at least three independent experiments. Statistical analyses were assessed by Student’s *t*-test (two variables). Statistical significance was established as *** *p* 0.001; ** *p* < 0.01; * *p* < 0.05.

## 3. Results

### 3.1. Comparison of the Effects of Novel Immunomodulatory mAbs and Clinically Validated Ones on hPBMCs Activation

**Figure 1:**
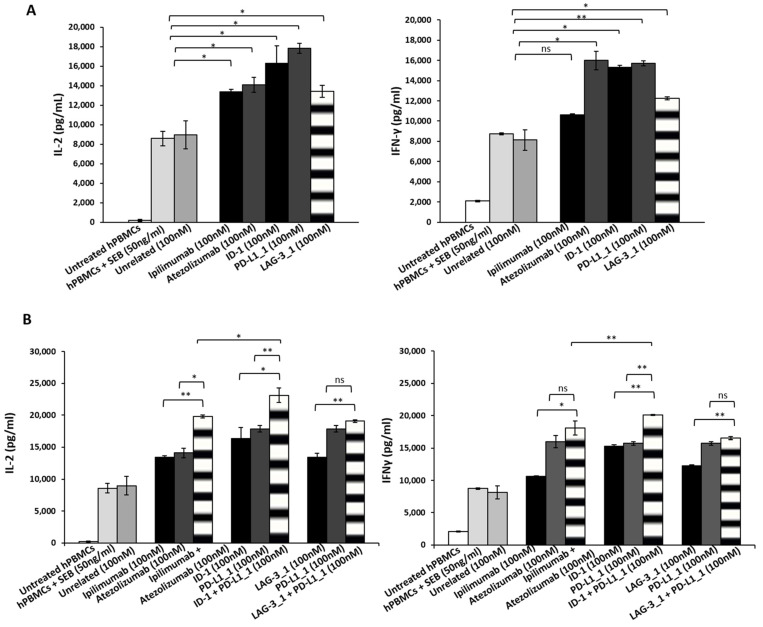
Effects of novel mAbs on the activation of stimulated lymphocytes. hPBMCs were stimulated for 66 h with SEB in the absence or in the presence of anti-CTLA-4 (black bars), anti-PD-L1 (dark grey bars) or anti-LAG-3 (striped bars) mAbs used alone (**A**) or in combination (**B**). In the panel B the levels of IL-2 and IFN-γ secreted were measured by ELISA (black bars for anti-CTLA-4 and anti-LAG-3 mAbs, dark grey bars for anti-PD-L1 and striped bars for combinations). Untreated hPBMCs or treated with an unrelated IgG were used as negative controls (white and grey bars, respectively). Error bars depict means ± SD. *p*-values for the indicated compounds are: ** *p* < 0.01; * *p* < 0.05.

Since immune checkpoints (ICs) represent key players in positively or negatively regulating T cell immune response, several biological drugs targeting ICs (ICIs), such as monoclonal antibodies (mAbs), have been developed and approved by Food and Drug Administration (FDA) for cancer therapy in the last decade [12,13]. The last advance in cancer immuno-therapy, based on ICIs, was to combine them in order to improve the anti-tumor efficacy [38]. In this regard, we compared the ability of the novel anti-PD-L1, anti-CTLA-4 or anti-LAG-3 mAbs (called PD-L1_1, ID-1 and LAG-3_1, respectively), previously generated in our laboratory by an innovative phage display strategy applied on live activated hPBMCs, as previously described [34], to that of clinically validated immunomodulatory mAbs (atezolizumab and ipilimumab), generated by using purified recombinant protein or transgenic HuMAb mice, respectively [39,40,41,42]. In particular, we tested their ability of activating lymphocytes when used alone or in combinatorial treatments. Firstly, we tested these antibodies for their effects on the activation and the proliferation of human lymphocytes, by detecting the levels of IL-2 and IFN-γ cytokines secreted in the supernatant, after the treatment with each mAb. To this aim, we stimulated human peripheral blood lymphocytes (hPBMCs) with Staphylococcal Enterotoxin B (SEB) and treated them for 66 h with PD-L1_1, ID-1, LAG-3_1 or the commercial atezolizumab or ipilimumab mAb, by using them as single agents (Figure 1A) or in appropriate combinations (Figure 1B) at the concentration of 100 nM. The negative controls were represented by untreated lymphocytes, hPBMCs stimulated with SEB or treated with an unrelated human mAb.

As shown in Figure 1A, the novel mAbs—with the exception of LAG-3_1—when used as single agents, activated hPBMCs more efficiently than the mAbs in clinical use. In particular, they showed increased levels of IFNγ with respect to ipilimumab and higher IL-2 secretion than both ipilimumab and atezolizumab. Furthermore, the combination of the novel immunomodulatory mAbs ID-1 and PD-L1_1 showed a significantly higher activation of lymphocytes, when compared to the combination of their corresponding anti-CTLA-4 (ipilimumab) and anti-PD-L1 (atezolizumab) mAbs in clinical use (Figure 1B), as evidenced by IL-2 secretion reaching levels of about 23,000 pg/mL.

### 3.2. Comparison of Cytotoxic Effects of Novel ICI mAbs and the Corresponding Ones in Clinical Use on Triple Negative Breast Cancer Cells

**Figure 2:**
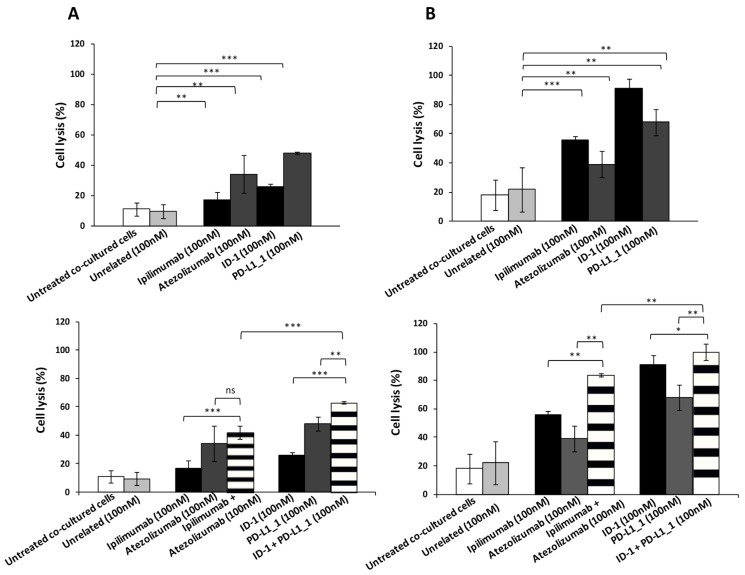
Cytotoxic effects of novel immunomodulatory mAbs on tumor cells co-cultured with hPBMCs. BT-549 (**A**) or MDA-MB-231 (**B**) tumor cells were co-cultured with hPBMCs (Effector:Target cells ratio 5:1) and treated for 48 h with anti-CTLA-4 (black bars) and anti-PD-L1 (dark grey bars) mAbs or their combination (striped bars) at the indicated concentrations. Co-cultured cells untreated or treated with an unrelated IgG were used as negative controls (white and grey bars, respectively). Cell lysis was measured by detecting LDH release, as described in Materials and Methods. Error bars depict means ± SD. *p*-values for the indicated compounds are: *** *p* ≤ 0,001; ** *p* < 0.01; * *p* < 0.05.

The expression of ICs, especially that of PD-L1, is reported in literature on several types of tumor cells, such as TNBC cells [43,44]. Their role in tumor cells is related to proliferative intracellular pathways and to the evasion of the immune response. With the aim of testing the immune response against TNBC, we investigated the effects of these novel immunomodulatory mAbs on cancer cells when co-cultured with hPBMCs, for mimicking in vitro the in vivo tumor environment. Thus, BT-549 or MDA-MB-231 TNBC cells were cultured in the presence of hPBMCs (Effector:Target cells ratio 5:1) and treated for 48 h with the novel ID-1 or PD-L1_1 mAbs at the concentration of 100 nM, used as single agents or in combination. The commercial anti-CTLA-4 and anti-PD-L1 mAbs (ipilimumab and atezolizumab), or their combination, were used as positive controls in parallel assays, whereas cells untreated or treated with an unrelated human mAb were used as negative controls. The cytotoxic effects induced by the treatments were detected by measuring the lactate dehydrogenase (LDH) release in the supernatant of co-cultures. As reported in Figure 2, the novel ID-1 and PD-L1_1 mAbs exerted cytotoxic effects on TNBC more efficiently than the corresponding FDA approved mAbs, particularly when tested on MDA-MB-231 cancer cells (Figure 2B), where they showed 91% and 68% of cell lysis respectively. Furthermore, their combination led to a further enhancement of cancer cell death, that was more marked than that observed in the combinatorial treatments of atezolizumab and ipilimumab, reaching even 100% of cell lysis when used on MDA-MB-231 cancer cells (Figure 2B).

**Figure 3:**
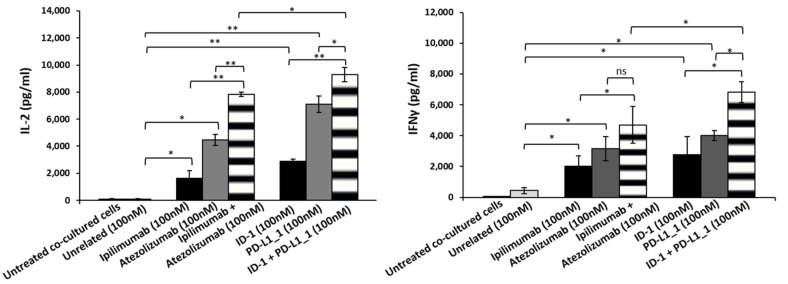
Effects of novel immunomodulatory mAbs on the secretion of IL-2 and IFN-γ by co-cultures of BT-549 tumor cells with hPBMCs. IL-2 and IFN-γ secretion levels were measured by ELISA on supernatants of cells treated with anti-CTLA-4 (black bars), anti-PD-L1 (dark grey bars) mAbs or their combination (striped bars) for 48 h. Cells untreated or treated with an unrelated IgG were used as negative controls (white and grey bars, respectively). Error bars depict means ± SD. *p*-values for the indicated compounds are: ** *p* < 0.01; * *p* < 0.05.

**Figure 4:**
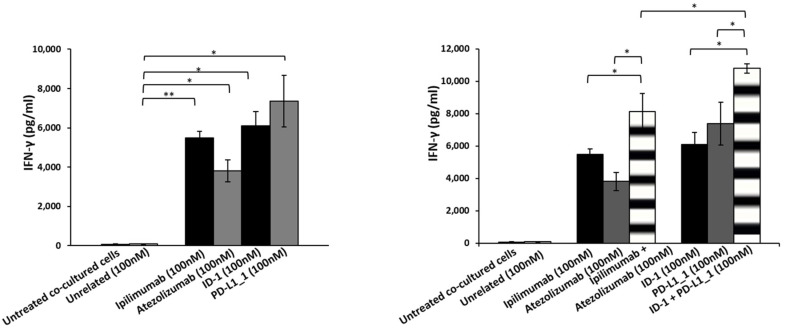
Effects of novel immunomodulatory mAbs on the secretion of IFN-γ in co-cultures of MDA-MB-231 tumor cells with hPBMCs. Levels of IFN-γ secreted by hPBMCs co-cultured with tumor cells and treated with anti-CTLA-4 (black bars) or anti-PD-L1 (dark grey bars) mAbs and their combination (striped bars) at the indicated concentration for 48 h. Cells untreated or treated with an unrelated IgG were used as negative (white and grey bars, respectively). Error bars depict means ± SD. *p*-values for the indicated compounds are: ** *p* < 0.01; * *p* < 0.05.

In order to clarify whether tumor cell lysis is correlated to the increased activation of lymphocytes, we measured the levels of cytokines, markers of hPBMCs proliferation and functionality, released in the supernatants of these co-cultures, by comparing the effects of the novel isolated mAbs to those of the commercial ipilimumab and atezolizumab, when used in combination. As shown in Figure 3 and Figure 4, IFN-γ and IL-2 levels were higher in the supernatants of co-cultures treated with the novel mAbs with respect to those observed in treatments with ipilimumab and atezolizumab. Specifically, ID-1 and PD-L1_1 doubled IL-2 levels, secreted by hPBMCs co-cultured with BT-549 cells, of their corresponding clinically validated mAbs. Combinatorial treatments of ID-1 and PD-L1_1 improved even more than single agent treatments the cytokines release, thus confirming that their higher anti-tumor activity is related to a more efficient activation of hPBMCs.

### 3.3. Comparison of the Cardiotoxic Side Effects of Novel mAbs with Those of the Clinically Validated Atezolizumab and Ipilimumab

Despite the considerable advantages of cancer immunotherapy with respect to conventional therapies; still, a wide spectrum of side effects have been reported [45,46,47]. Among the more severe adverse events, some cases of myocarditis and pericarditis have been evidenced especially in combinatorial treatments of anti-ICs mAbs [32,33].

In order to evaluate the safety of the novel PD-L1_1 and ID-1 mAbs, we investigated their effects on human fetal cardiomyocytes (HFCs) co-cultured in presence of hPBMCs, and compared their anti-tumor effects with eventual cardiac side effects. We used human fetal cardiomyocytes as we previously evidenced the expression of the target immune check points (PD-L1 and CTLA-4) [15] recognized by the immunomodulatory mAbs, as previously reported in literature also for adult human and murine heart [48]. Furthermore, HFC can be cultured and used routinely differently from adult cardiomyocytes that should be collected from patients and are not easily kept in culture.

**Figure 5:**
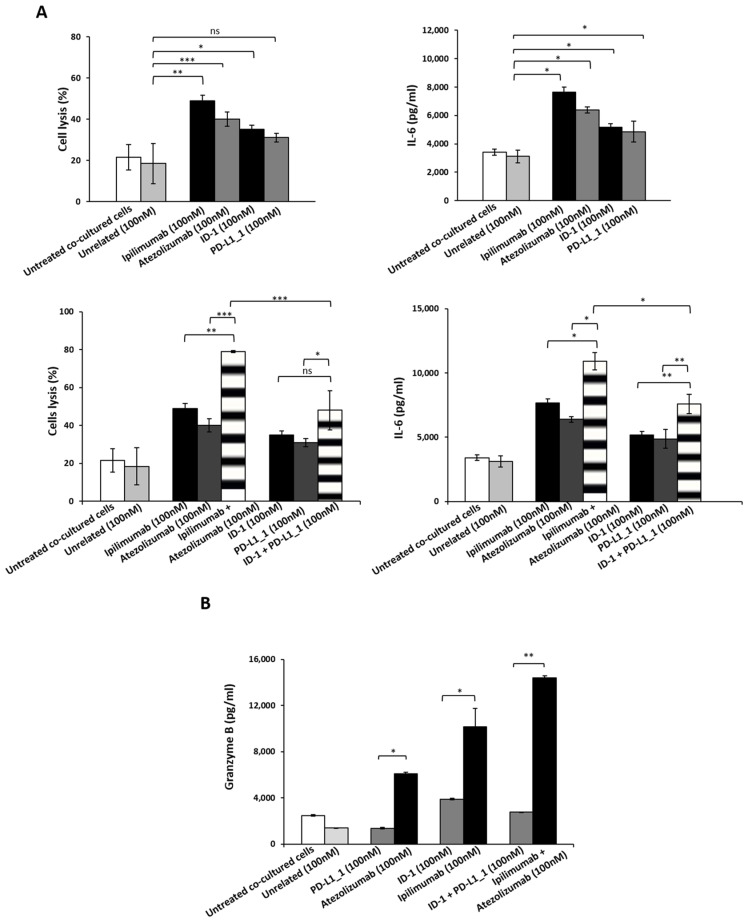
Cardiotoxic and pro-inflammatory effects induced by immunomodulatory mAbs or their combination on human fetal cardiomyocytes. (**A**) Cardiotoxicity of novel immunomodulatory mAbs was analyzed by detecting the LDH released by HFC cells co-cultured with hPBMCs in the absence or presence of single mAbs (black and grey bars) or their combinations (striped bars) for 24 h. The pro-inflammatory effects of the indicated immunomodulatory mAbs were analyzed by evaluating the secretion of IL-6 in the supernatant of the co-cultures treated with anti-CTLA-4 (black bars), anti-PD-L1 mAbs (dark grey), or their combination (striped bars). IL-6 is expressed as pg/mL. (**B**) The release of Granzyme B was evaluated by detecting its levels in the supernatant of co-cultures treated as indicated. As negative controls, cells untreated or treated with unrelated IgG were used (white and grey bars, respectively). The values were reported as the mean of at least three determinations obtained in three independent experiments. Error bars depict means ± SD. *** *p* ≤ 0,001; ** *p* < 0.01; * *p* < 0.05.

To this aim, HFC were co-cultured with hPBMCs (Effector:Target ratio 5:1) and treated with PD-L1_1, ID-1 or their combination for 24 h at the concentration of 100 nM. In parallel assays, we tested the cardiotoxic effects of the FDA approved mAbs specific for the same targets (atezolizumab and ipilimumab), used as positive controls and co-cultures untreated or treated with an unrelated human mAb, as negative controls. As shown in Figure 5, treatments with PD-L1_1 or ID-1 mAbs induced much lower cardiac cell lysis with respect to ipilimumab or atezolizumab mAbs. Moreover, the levels of IL-6, a proinflammatory cytokine frequently associated with cardiac injury [32,33], turned out to be lower when the cells were treated with the novel immunomodulatory mAbs with respect to those observed in treatments with ipilimumab and atezolizumab. In addition, the combination of the commercial ipilimumab and atezolizumab was found to induce higher cardiac cell lysis (about 80%) with respect to the combination of ID-1 and PD-L1_1 (about 50%). In line with these data also IL-6 secretion resulted higher when the cardiomyocytes were treated with the clinically validated mAbs with respect to the corresponding novel mAbs. Similar results were also observed when we compared the levels of TNF-α, another well-known inflammatory cytokine, released in the supernatant of co-cultures after the treatments (data not shown). By ELLA Simple Plex assay, we detected TNF-α levels in the supernatant of co-cultures of cardiac cells and hPBMCs and we measured in the combination of the commercial ipilimumab and atezolizumab, a value of about 7000 pg/mL of TNF-α, compared to that of about 1000 pg/mL, detected in the combinatorial treatment with the novel mAbs.

To further investigate the side effects of the novel mAbs with respect to those of ipilimumab and atezolizumab, we also evaluated the levels of Granzyme B, usually released by either cytotoxic CD8+ T cells or by natural killer (NK) cells to eliminate tumor cells. In agreement with the previous results, we found a significant increase of Granzyme B in the supernatant of co-cultures of HFC and hPBMCs treated with atezolizumab, ipilimumab, or their combination, whereas no or very low effects were observed in treatments with ID-1, PD-L1_1 or their combination. Specifically combinatorial treatments with the FDA approved mAbs led to Granzyme B secretion levels of about 14,400 pg/mL, with respect to that of about 3000 pg/mL observed in the treatment with ID-1 plus PD-L1_1.

These in vitro findings indicate that the novel mAbs are endowed with higher anti-tumor efficacy and lower cardiotoxic side effects with respect to ipilimumab and atezolizumab.

**Figure 6:**
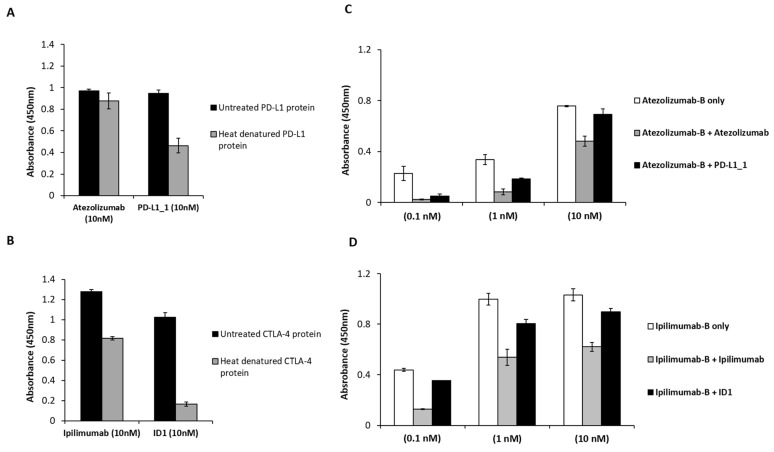
Binding assays to define the epitopes recognized by the novel mAbs. ELISA assays performed on PD-L1/Fc (**A**) or CTLA-4/Fc (**B**) before and after heat denaturation treatment of immobilized proteins. Competitive ELISA assays were performed by testing the binding of the biotinylated antibodies atezolizumab (**C**) or ipilimumab (**D**) to PD-L1 or CTLA-4 chimeric proteins in the absence (white bars) or presence of molar excess of unlabeled competitive PD-L1_1 or ID-1 antibodies (black bars). As positive controls biotinylated atezolizumab or ipilimumab (grey bars) were used in the presence of their unlabeled counterparts. Binding values were reported as the mean of at least three determinations obtained in three independent experiments. Error bars depicted means ± SD.

To explain the reason why ipilimumab and atezolizumab mAbs induced lower cytotoxic effects on cancer cells and a higher cardiotoxicity on HFC cells than those obtained with the novel generated mAbs, we hypothesized that the novel ID-1 and PD-L1_1 mAbs could exert different effects by recognizing different epitopes with respect to those of the clinically validated mAbs. Therefore, we performed ELISA assays to test the binding ability of each mAb for its target before and after denaturation of the immobilized target protein to investigate on their preferential ability to recognize a conformational epitope, considering that ID-1 and PD-L1_1, differently from atezolizumab and ipilimumab, were selected on live lymphocytes. To this aim, after coating, the immobilized proteins were incubated at 65 °C for 30 min or left at room temperature before the blocking and the following steps of ELISA assays (see Methods). As shown in Figure 6A,B, when PD-L1/Fc or CTLA-4/Fc were denatured, the binding of the novel mAbs was significantly reduced, thus suggesting that PD-L1_1 and ID-1 preferentially recognize the protein targets in their native conformation. On the contrary, the clinically validated atezolizumab and ipilimumab were able to recognize both the native and denatured proteins with comparable affinities, suggesting that they could recognize linear epitopes.

To further investigate on the hypothesis of different epitopes, we also performed competitive ELISA assays by measuring the binding to immobilized PD-L1 or CTLA-4 protein of biotinylated atezolizumab and ipilimumab, in the absence or in the presence of saturating concentrations of unlabeled PD-L1_1 or ID-1 mAb, respectively. As a control, each biotinylated mAb was tested also in the presence of a molar excess of its unlabeled counterpart. As shown in Figure 6C,D, the binding abilities of biotinylated atezolizumab or ipilimumab to their targets in the presence of PD-L1_1 or ID-1 mAb were only slightly inhibited compared to their unlabeled counterparts, thus suggesting that the novel anti-PD-L1 and anti-CTLA-4 mAbs bind to distinct but overlapping epitopes with respect to those recognized by the FDA approved mAbs.

### 3.4. Evaluation of Combinatorial Treatments including LAG-3_1

**Figure 7:**
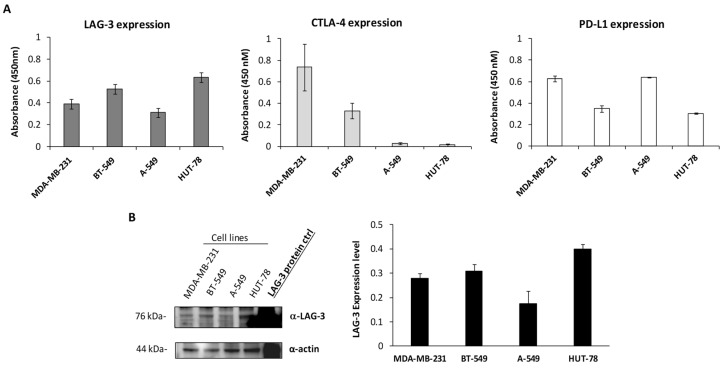
Expression of PD-L1, PD-1, CTLA-4, and LAG-3 ICs on a panel of cancer cells. (**A**) Cell ELISA assays were performed on MDA-MB-231, BT-549, A-549, or HuT-78 tumor cells to measure the cell surface expression of the indicated ICs with commercial anti-PD-L1, anti-CTLA-4, or anti-LAG-3 mAbs. Binding values were reported as the mean of at least three determinations obtained in three independent experiments. (**B**) Western blotting analyses of extracts from MDA-MB-231, BT-549, A-549, and HuT-78 tumor cells, obtained by using the commercial anti-LAG-3 mAbs. The intensity of the bands corresponding to ICs was normalized to actin (see also Table S1).

In order to choose appropriate target cells, to test the combinations of ICIs including LAG-3_1, we measured by cell ELISA and Western blotting the expression of LAG-3 on a panel of different cell lines. As shown in Figure 7A and Figure S1, the TNBC cell lines express high levels of PD-L1 and CTLA-4, but low levels of LAG-3, whereas the HuT-78 cells, derived from cutaneous T cell Lymphoma, express high levels of LAG-3. Thus, we chose to use these cells to better investigate the role of the anti-LAG-3 antibody used alone or in combination with PD-L1_1. However, in parallel assays, we tested also their effects on TNBC MDA-MB-231 cells. As expected, considering the low level of LAG-3 expression on MDA-MB-231 cells, the effects of LAG-3_1 on the latter cells in co-cultures with hPBMCs was limited (about 20% of cells lysis), but reached almost 40% when it was combined with PD-L1_1. Accordingly, the level of IFN-γ released in the treated cells with LAG-3_1 was about 3000 pg/mL when it was used alone and reached about 12,000 pg/mL when combined with PD-L1_1.

**Figure 8:**
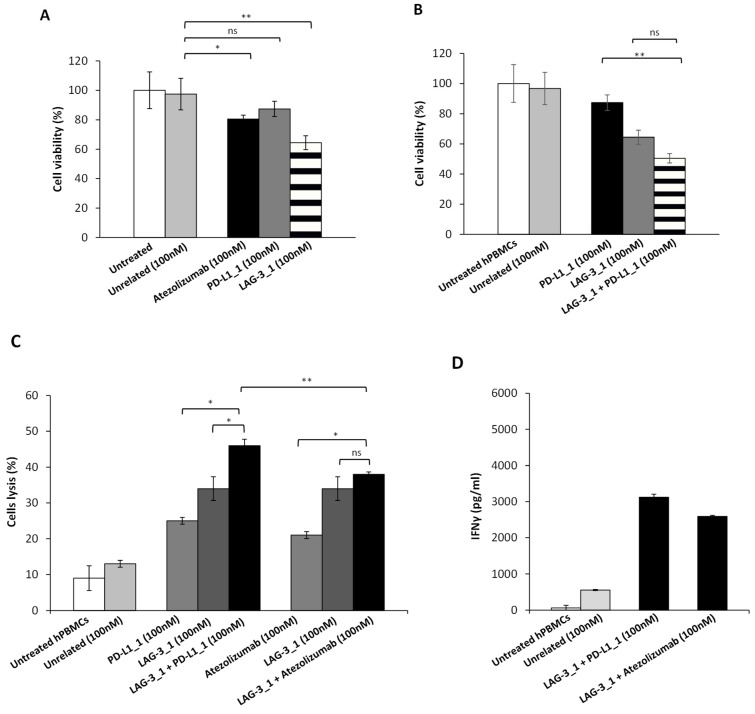
Effects of novel immunomodulatory mAbs on viability of HuT-78 tumor cells in the absence of lymphocytes. (**A**,**B**) MTT assays were performed on the supernatant of HuT-78 tumor cells, treated for 72 h with anti-PD-L1 or anti-LAG-3 mAbs, used as single agents or in combinatorial treatments. Cell viability is expressed as percentage of viable cells with respect to control untreated ones. Error bars depict means ± SD ** *p* < 0.01; * *p* < 0.05. (**C**) LDH levels were detected in the supernatant of HuT-78 tumor cells co-cultured with hPBMCs (Effector:Target cells ratio 5:1), treated for 48 h with anti-PD-L1 or anti-LAG-3 mAbs, or their combination, at the indicated concentrations. (**D**) Levels of IFN-γ secreted by hPBMCs co-cultured with tumor cells and treated with anti-CTLA-4, anti-PD-L1, or anti-LAG-3 mAbs, or their combination, at the indicated concentration for 48 h. Cells untreated or treated with an unrelated IgG were used as negative controls. Error bars depict means ± SD. *p*-values for the indicated compounds are: ** *p* < 0.01; * *p* < 0.05.

We then tested the effects of PD-L1_1 and LAG-3_1 on HuT-78 cell line expressing higher levels of LAG-3. To this aim, we first treated these cells with PD-L1_1 or LAG-3_1 mAbs, or their combination, for 72 h and then measured their cell viability in the absence of hPBMCs. Atezolizumab, or its combination with LAG-3_1, were used in parallel assays as positive controls, whereas cells untreated or treated with a human unrelated mAb were used as negative controls. As reported in Figure 8A, when used as single agents, both PD-L1_1 and LAG-3_1 significantly inhibited tumor cell growth (Figure 8B). More interestingly, the combination of LAG-3_1 and PD-L1_1 showed additive effects reaching about 50% of tumor growth inhibition, thus suggesting that also this combination could be useful to achieve stronger anti-tumor activity than that observed with single agent treatments.

In order to investigate on the effects of anti-PD-L1 or anti-LAG-3 mAbs on HuT-78 in the presence of lymphocytes, we co-cultured tumor cells with hPBMCs (Effector:Target cells ratio 5:1) for 48 h and treated them with atezolizumab, PD-L1_1, LAG-3_1, or their combinations. Co-cultures untreated or treated with an unrelated mAb were used as negative controls. As shown in Figure 8C, PD-L1_1 mAb was found to be more cytotoxic than the FDA approved atezolizumab, whereas LAG-3_1 showed the highest effects among the single agent treatments. Furthermore, combinatorial treatments induced a significant higher tumor cell lysis with respect to single agent treatments and the combination involving LAG-3_1 and the novel PD-L1_1 mAb showed higher efficacy (reaching 48% of cell lysis), with respect to the one involving atezolizumab (38% of cell lysis). Accordingly, the levels of the pro-inflammatory IFN-γ cytokine released by hPBMCs when co-cultures were treated with the combination of the novel mAbs LAG-3_1 and PD-L1_1 resulted higher than that observed with the combinatorial treatment involving the FDA approved atezolizumab. Furthermore, in addition to the satisfactory anti-tumor activity observed on T cell lymphoma, low or not significant cardiotoxic side effects were observed when the combination of LAG-3_1 and PD-L1_1 was tested on cardiomyocytes co-cultured with hPBMCs (data not shown).

## 4. Discussion

TNBC is characterized by stromal and intratumoral infiltration of lymphocytes [49], associated with high levels of ICs expression, such as that of PD-L1 [44,50], which led to FDA approval in 2019 of the anti-PD-L1 mAb, atezolizumab, in combination with paclitaxel for the treatment of metastatic TNBCs [10] even though the following IMpassion131 trial (NCT03125092) caused its withdrawal [11].

In this study, we tested the effects of the novel human immunomodulatory mAbs targeting PD-L1, CTLA-4, and LAG-3 on TNBC and T-cell lymphoma derived cells in comparison with the clinically validated atezolizumab and ipilimumab, by using them as single agents or in combinations, in order to test eventual synergistic or additive effects.

To this aim, the novel immunomodulatory mAbs (PD-L1_1, ID-1 and LAG-3_1), previously generated in our laboratory by a phage display selection on activated lymphocytes [34], were analyzed by evaluating their ability to activate stimulated hPBMCs. The novel ID-1 and PD-L1_1 mAbs showed potent lymphocyte activation, inducing the secretion of higher levels of IL-2 and IFN-γ cytokines with respect to atezolizumab and ipilimumab, either when used as single agents or in combination.

In order to identify the combinatorial treatments endowed with the most powerful anti-tumor effects and the lowest side effects, we then tested these mAbs on in vitro models based on co-cultures of hPBMCs with either tumor cells or human fetal cardiomyocytes. The latter choice derived from the recent reports on the rare but severe cases of myocarditis [32], induced by combinatorial treatments including ipilimumab.

We demonstrated that the novel anti-PD-L1 and anti-CTLA-4 immunomodulatory mAbs used as single agents were able to induce higher cytotoxic effects on some TNBC cell lines than the FDA clinically validated mAbs. The efficient tumor cell lysis induced by these mAbs was explained by the increased activation of lymphocytes co-cultured with tumor cells, leading to enhanced levels of IFN-γ and IL-2 release, with respect to those treated with atezolizumab and ipilimumab. More interestingly, the treatments with the novel immunomodulatory mAbs induced lower cytolysis of human cardiomyocytes co-cultured with hPBMCs, with respect to those observed with the clinically validated ipilimumab and atezolizumab, accompanied by lower levels of IL-6, a pro-inflammatory cytokine involved in the etiopathogenesis of myocarditis [32,33]. Further evidence of the reduced side effects of the novel immune checkpoint inhibitors was provided by lower levels of granzyme B, as well as those of TNF-α, detected in the supernatants of treated HFC co-cultured with hPBMCs, compared to those measured in the supernatants of treatments with atezolizumab and ipilimumab.

Thus, the parallel co-cultures-based assays allowed us to early compare the efficacy and safety of ipilimumab plus atezolizumab and PD-L1_1 plus ID-1 combinations in order to identify the most potent anti-tumor drugs with the lowest cardiotoxic side effects. Indeed, PD-L1_1 plus ID-1 treatment showed potent effects on tumor cells lysis, reaching 100% of lysis in co-cultures of MDA-MB-231 cells and inducing a higher secretion of IL-2 and IFN-γ with respect to atezolizumab and ipilimumab, but exerted opposite effects on cardiomyocytes.

Similar interesting results were observed also with the combination of LAG-3_1 and PD-L1_1 that showed low cytotoxic effects on HFC and satisfactory anti-tumor activity also on T cell lymphoma-derived cells.

The differential effects of the novel anti-CTLA-4 and anti-PD-L1 mAbs, with respect to ipilimumab and atezolizumab could be explained by considering the distinct although overlapping epitopes recognized by the two mAbs, as highlighted by experimental evidence provided here and confirmed by the previous reports on their different mechanisms of action [51]. Indeed, the novel antibody ID-1 was found able to inhibit cell growth by inducing a marked phosphorylation of CTLA-4, inhibiting the phosphorylation of protein kinase B (PKB/Akt) and inducing caspase activation differently from ipilimumab. Similarly, PD-L1_1 mAb affected intracellular pathways downstream PD-L1 in tumor cells by inhibiting the phosphorylation of Erk, JNK, and P38 in a different fashion with respect to atezolizumab which did not affect the level of p-JNK, and showed only a slight effect on p-P38 [14]. Furthermore, the two anti-PD-L1 mAbs could have differential properties related also to their different isotypes, with PD-L1_1 an IgG4 being devoid of effector functions in comparison with the IgG1 format of atezolizumab.

Altogether, these findings indicate that the novel antibodies could be useful tools for therapeutic approaches in TNBC lacking cardiotoxic side effects.

## 5. Conclusions

We report here that novel anti-CTLA-4 and anti-PD-L1 immunomodulatory mAbs, previously generated in our laboratory, have greater anti-tumor activity than the clinically validated ipilimumab and atezolizumab on TNBC cells, well known for their aggressiveness and resistance to conventional chemotherapeutic treatments. In particular, the novel ID-1 and PD-L1_1, used as single agents or in combination, not only induced tumor cell lysis by activating hPBMCs more efficiently than the FDA approved mAbs, but also showed lower cardiotoxic side effects.

In addition, we show that the combination of a novel anti-LAG-3 mAb with PD-L1_1 have remarkable effects also on T cell lymphoma derived cells. Thus, we conclude that the use of co-cultures based assays on tumor and cardiac cells could be useful to choose the best combinatorial treatments and to early predict both anti-tumor efficacy and lack of cardiotoxic side effects of immunomodulatory mAbs.

## Data Availability

The data presented in this study are available in this article (and Supplementary Material).

## Supplementary Materials

The following are available online at www.mdpi.com/article/10.3390/cancers14010121/s1, Figure S1: Full length blots of Figure 7B, Table S1: Analysis by densitometry of Western Blot of Figure S1 (relative to Figure 7B).
