# Supplementary material for: Novel Combinations of Human Immunomodulatory mAbs Lacking Cardiotoxic Effects for Therapy of TNBC
Source: Cancers (Basel). 2021 Dec 27;14(1):121. doi: 10.3390/cancers14010121 (PMC8750931; doi:10.3390/cancers14010121)
Supplement: Supplementary file 1 [file cancers-14-00121-s001.zip › cancers-1519889-supplementary.pdf]

Supplementary Materials

Novel Combinations of Human Immunomodulatory mAbs Lacking Cardiotoxic Effects for Therapy of TNBC

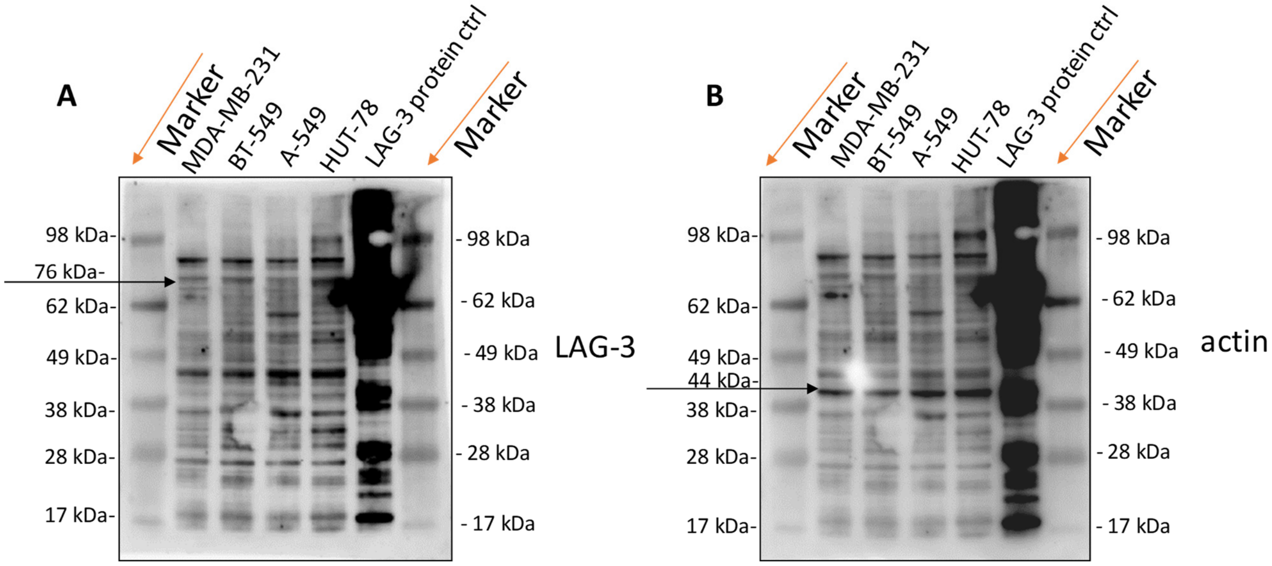

Figure S1. Full length blots of Figure 7B. (**A**) Western blot for detection of LAG-3 levels in the indicated cell extracts: the full lenght filter was stained with an anti-LAG-3 mAb. (**B**) Western blot for detection of actin levels in the indicated cell extracts: the same filter was then stained with an anti-Actin mAb. The intensity of the bands was normalized to actin. A marker for M.W. reference (17, 28, 38, 49, 62 and 98 kDa) was included.

Table S1. Analysis by densitometry of Western Blot of Figure S1 (relative to Figure 7B). Intesity signals of the bands were calculated by Image Lab software.

	MDA-MB-231	BT-549	A-549	HuT-78
LAG-3 protein densitometry readings	31057	33490	31852	80970
ACTIN protein densitometry readings	111155	108898	180998	203184
Intensity Ratio of LAG-3/ACTIN	0,2794	0,30754	0,17598	0,39851
